# A Case of Congenital Hypotonia and Developmental Delay in an Individual with a *De Novo* Variant Outside of the Canonical HX-Motif of ATN1

**DOI:** 10.1155/2023/1581876

**Published:** 2023-01-10

**Authors:** Elizaveta Makarova, Nicole R. Legro, Ermal Aliu

**Affiliations:** ^1^The Pennsylvania State University College of Medicine, Hershey, PA, USA; ^2^Penn State Health Milton S. Hershey Medical Center, Hershey, PA, USA; ^3^Department of Pediatrics, Division of Medical Genetics, Hershey, PA, USA

## Abstract

We present a case of a 4-year-old female with a de novo heterozygous variant in the ATN1 gene. The whole exome sequencing was performed on the patient and her parents, and a likely pathogenic, de novo variant was identified in exon 5 of the ATN1 gene. There are two well-documented conditions associated with the ATN1 gene: congenital hypotonia, epilepsy, developmental delay, and digital anomalies (CHEDDA) syndrome and dentatorubral-pallidoluysian atrophy (DRPLA). Unlike DRPLA which is caused by an expanded trinucleotide repeat, CHEDDA syndrome is caused by variants in the histidine-rich (HX) motif at exon 7 of ATN1 similar to the de novo variant found in exon 5 of the presented individual. CHEDDA syndrome is a neurodevelopmental disorder previously documented in over 17 unrelated individuals. Compared to other documented CHEDDA syndrome cases, this individual shares similarities in respect to hypotonia, hearing impairment, impaired gross and fine motor ability, gastrointestinal abnormalities, hyperextensible joints, and frontal bossing. However, the individual presented here has only a moderate developmental delay and has acquired more developmental milestones such as higher-level language skills and more developed fine motor skills, than previously described individuals. The authors of this paper believe the patient's milder phenotype may be due to the variant's location outside of the canonic HX motif.

## 1. Introduction

ATN1 encodes an evolutionarily conserved and highly expressed protein, atrophin-1 (OMIM# 607462). The gene is located on chromosome 12 (12p13.31) and encodes two transcripts which translate to the same protein. Atrophin-1 functions as a nuclear transcriptional corepressor that has important roles in nuclear signaling, particularly embryonic organ development [1]. Early studies in drosophila demonstrated atrophin-1 to be required for early embryonic patterning as a key regulator of the canonical “even-skipped” protein in drosophila development [2]. Regarding human brain development, atrophin-1 has been shown to interact with and modulate several key proteins critical for neural progenitor cell survival, proliferation, and neuronal migration [3]. However, the complete physiologic function of ATN1 in the human body is still unknown. The protein has several functional domains in which perturbations have been linked to two human diseases.

Pathogenic trinucleotide expansions of exon 5 have been documented and lead to disruptions in the polyglutamate tract of the protein and cause dentatorubral-pallidoluysian atrophy (DRPLA) (OMIM# 125370). DRPLA is a progressive neurodegenerative disease which clinically resembles Huntington's disease with overlapping features of ataxia, seizures, choreoathetosis, and dementia [4]. Similarly, the pathogenesis of DRPLA involves intranuclear inclusions in neurons and glial cells related to the novel biochemical properties of the errant protein [5].

Palmer et al. [6] described a new clinical entity, CHEDDA syndrome, caused by de novo missense or insertion/deletion/duplication variants exclusively located in exon 7 of the gene (OMIM# 618494). The acronym describes key findings of the syndrome: congenital hypotonia, epilepsy, developmental delay, and digital anomalies, which have been described in 17 individuals to date [6, 7]. Other findings include congenital anomalies of the heart, kidneys, and vertebrate; malformations of the brain; and feeding difficulties [6, 7]. Functional characterization of the disorder revealed that the variants disrupted the HX motif of the protein (histidine plus any other amino acid repeated eight times). The region is hypothesized to confer critical pH-dependent binding properties for ions or other small molecules that interact with atrophin-1 [7].

## 2. Case Presentation

We report the case of a 4-year-old female who presented to pediatric genetic specialists with congenital hypotonia, bilateral conductive hearing loss, hyperextensible joints, and “staring spells.” The individual was 3.2 kilograms at 36 weeks by repeat *C*-section to a mother 33 years of age, gravida 2, para 2. The individual experienced transient tachypnea postnatally and was admitted to the NICU for 7 days due to respiratory distress, where she required supplemental oxygen. Patient passed her initial newborn hearing screening test. Due to tongue thrusting when food or a spoon was placed in the mouth, patient underwent frenectomy at the age of 3 months. The patient failed her 5-month hearing screening, leading to an evaluation by otolaryngology, which noted bilateral conductive hearing loss.

At 10 months, the patient presented with mild hypotonia, hyperextensible joints, and isolated patellar hyperreflexia. She had relative macrocephaly (head circumference in 98^th^ percentile) and frontal bossing. She was reaching normal developmental milestones with the ability to crawl and pull herself up from sitting to standing. She, however, had started puree and Step 1 foods later than expected. At 16–17 months, she experienced frequent ear infections. She presented at 20 months with developmental delay and continued hypotonia. She experienced a leg length discrepancy and dragged her left foot, refusing to lift it. She required speech and physical therapy. The patient also slept in 3–5 hour stretches and woke frequently.

At 22 months, she was experiencing right leg weakness that accompanied her turning her right foot in when walking, toe walking on her right side, and falling a lot. She continued to have macrocephaly with frontal bossing even after her fontanelles closed. At 2 years old, it was noted she had poor dexterity in her fingers, could run but tripped a lot, and continued to turn her foot in and drag it while walking. At this time, she began to have staring spells 1–2 times a week.

At 3 years old, she began stuttering while speaking and experiencing unresponsiveness in the middle of conversations. While talking, she would stop and stare, then go back to what she was saying as if nothing had happened. These events occurred about two times a day, lasting 5–10 seconds each time. The patient wore orthotics and a hip brace; her right leg was bigger than her left, and her physical therapist reported a missing gluteal crease. Her speech development was delayed; she was able to form 3–4-word single sentences, with about 45% of what she said understandable to a stranger. Her parents have noticed she fatigues easily for her age and has a low attention span. She also experiences chronic constipation and passes stool two times a week. She has some feeding difficulty due to oropharyngeal dysphagia. She has one café au lait spot on her right buttock.

Several imaging studies have been conducted. Brain CT imaging concluded prominent symmetrical ventricles. An MRI of the brain found mild lateral ventriculomegaly without evidence of decompensated or obstructed hydrocephalus. An electroencephalogram (EEG) study was performed and deemed normal for the patient's age. Echocardiography showed normal intracardiac situs relationships, anatomy, and function, as well as normal chamber size and biventricular systolic function. Her renal ultrasound was normal. An MRI of the lumbar spine was determined to be normal by radiology.

**Figure 1:**
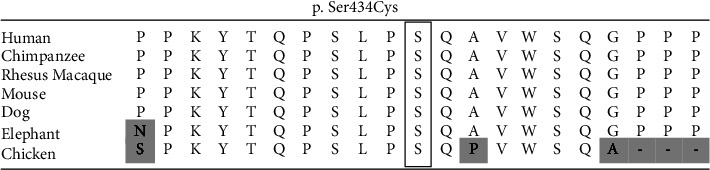
Results of sequence alignment for atrophin-1 surrounding amino acid residue 434 in seven species including *Homo sapiens*, *Pan troglodytes*, *Macaca mulatta*, *Mus musculus*, *Canis lupus familiaris*, *Loxodonta africana*, and *Gallus gallus*.

The whole exome sequencing was performed on the patient, at the age of 3, and her parents (XomeDxPlus, GeneDx). Results identified a de novo heterozygous variant in exon 5 of ATN1 (c.1301 C > G, p. Ser434Cys) predicted to be likely pathogenic. The variant was absent in both parents. The variant was classified as likely pathogenic based on the commercial testing, and several in silico prediction tools have predicted the variant to be likely pathogenic as well (Table 1). A multiple sequence alignment of seven species shows the variant in question occurs in a highly conserved residue (Figure 1).

## 3. Discussion

ATN1 has been mapped to the short (p) arm of chromosome 12 and consists of 11 exons. Atrophin-1 includes a histidine-rich motif, a serine repeat region, and variable glutamine repeat sections. The protein has low tissue and brain region specificity. Atrophin-1 is expressed in nervous tissue and can be found in the nuclear and cytoplasmic compartments of the neurons [13]. The gene is known to play a role in the development of the nervous system. Atrophin-1's functional properties include recruiting NR2E1 to repress transcription and associating with nuclear receptors, forming complexes, and regulating transcription [13].

CHEDDA syndrome variants are most often missense mutations but also include insertions, deletions, and duplications within a 16-amino-acid sequence of exon 7. This is in contrast with our presented case, where the variant is located within exon 5 and outside of the well-described polyglutamate tract and HX repeat regions of the protein that have been associated with human disease. There are no significant differences in clinical presentation between previously documented cases in which the variant is a missense mutation compared to an insertion, deletion, or duplication. The presented case shares distinct characteristics with the other 17 documented cases of CHEDDA syndrome (Table 2). The previously documented cases already documented a spectrum of phenotypes, and our case presents with a milder phenotype, especially with respect to verbal, gross, and fine motor skills (Table 3). Palmer et al. [7] suggested alterations in the histidines in HX motifs affect the critical functioning of the protein, as patients with pathogenic variants outside the HX motif in the RERE gene (OMIM #605226) are less likely to have congenital anomalies in the brain, eye, and heart. Similarly, based on our single case, it is possible this pathomechanism applies to the ATN1 gene.

ATN1 has a CAG trinucleotide repeat that ranges from six to 35 repeats. If the repeat exceeds 35, there can be a gain of function mutation causing aggregation to occur in the nucleus, leading to an increase in cellular toxicity [13]. This trinucleotide expansion in exon 5 is associated with dentatorubral-pallidoluysian atrophy (DRPLA). DRPLA often presents with cerebellar ataxia, myoclonus, seizures, choreoathetosis, and dementia, with a mean age of onset of 30 years [14]. Our presented case shares the variant location of exon 5 but does not share the symptoms, age of onset, progressive nature, and lacks the mechanism of genetic variation and pathogenesis. One limitation of our study is the lack of functional studies, samplesize, and poorly understood pathomechanism. While loss of function intolerancehas been suggested for ATN1 by The Genome Aggregation Database (gnomAD v2.1.1browser with pLoF exp: 40.0, obs: 3, pLI = 1.00, o/e = 0.07) and Decipher(Predicted score for haploinsufficiency v.3 - 0.702926343), it is unclear ifthis is indeed the mechanism or a gain of function in a specific domain/pathway,and needs to be further explored.

In summary, the case presented has symptoms and a missense mutation similar to other reported cases of CHEDDA syndrome; however, it is located in exon 5 and not in the HX motif of exon 7. The patient does not share similarities in symptoms, age of onset, or type of DNA variance with DRPLA but is located in exon 5, similar to other reported cases of the atrophy. With the presentation of this case, we hope to describe the phenotype of an individual with a missense mutation in exon 5 of ATN1 that presents with milder characteristics compared to other described CHEDDA cases. Additional research is necessary to develop a better understanding of ATN1's role in neurodevelopment and regulation.

## Figures and Tables

**Table 1:** In silico prediction tool results for c.1301 C > G (p.S434C) variant [8–12].

Software	Prediction for c.1301C > G (p.S434C) variant
PolyPhen-2	0.999 (probably damaging)
Mutation taster	Disease causing
SIFT	Intolerant
MutPred2	0.198 (threshold 0.5)
PROVEAN	−2.010 (neutral)

**Table 2:** Comparison of presence/absence of features in previously documented cases of CHEDDA syndrome and presented case [6, 7].

	Presented case	CHEDDA syndrome
Facial gestalt	+	17/17
GDD or ID	+	16/17
Digital anomalies	−	14/17
Visual impairment	−	14/17
Musculoskeletal disorder	+	13/17
Respiratory symptoms	−	12/17
Hearing impairment	+	10/17
Seizures	?	9/17
Genitourinary disease	−	9/17
Congenital heart disease	−	8/17
Orofacial clefting	−	3/17

**Table 3:** Descriptive comparison of clinical and phenotypical features between the presented case and previously documented cases of CHEDDA syndrome including number of cases documented with each feature [6, 7].

	Presented case	CHEDDA syndrome
Variant	Missense point mutation	Missense point mutation (11)
		Deletion (3)
		Insertion (2)
		Duplication (1)

Antenatal concerns	None	None (10)
		Multiple fetal anomalies (2)
		Polyhydramnios (1)
		Small for gestational size (1)
		Increased nuchal translucency (1)
		Oligohydramnios (1)
		Cardiac malformation (1)

Current neurology	Global hypotonia	Global hypotonia (9)
		Central hypotonia and appendicular spasticity (3)
		Axial hypotonia and appendicular hypertonia (3)
		Reduced tone (1)
		Normal tone (1)

Verbal ability	Forms sentences of 3-4 words	Nonverbal (10)
		Babbles/coos (4)
		Few words (3)

Gross motor ability	Can walk, run, climb stairs; trips frequently when doing so	Cannot roll/immobile (5)
		Sits with support (5)
		Can roll/crawls (3)
		Stands with support (2)
		Walking (2)

Fine motor ability	Grasping; developing pincer grasp	Grasping (6)
		Limited/not grasping (3)
		Not reported (8)

GI abnormalities	Dysphagia	Poor feeding/dysphagia (11)
		GERD (7)
	Constipation	Constipation (6)
		None (1)

## Data Availability

The data are available upon request from the corresponding author upon request.
